# 

**Published:** 1871-04

**Authors:** 


### PERIODICALS AND BOOKS.

LYCEUM BANNER, published by E. H. Kimball, at 137½ Madison Street, Chicago, Ill., at one dollar a year, is an excellent little monthly for children.

THE HERALD OF HEALTH.—This is a very popular health magazine, published monthly, by Wood & Holbrook, 13 & 15 Laight Street, New York, at two dollars per annum. We will furnish it, with the BISTOURY, at the same price.

THE AMERICAN STOCK JOURNAL.—A very useful journal for stock breeders and farmers. Contains a vast amount of useful information. Is published by N. P. Boyer & Co., at Parkersburg, Pa., for one dollar a year. We will also furnish this journal with the BISTOURY, at the same price—one dollar.

AN EXCELLANT MEDICAL JOURNAL.—"The Medical Times," a semi-monthly journal of medical and surgical science, published by J. B. Lippencott & Co., of Phil., at four dollars a year. It is the best printed, and best edited medical journal in America. Send for a specimen copy.

"GOOD HEALTH."—A first class health journal for the people. Ably edited by the best medical writers of Boston—issued monthly, at two dollars a year, by Alexander Moore, 11 Broomfield Street, Boston. By sending two dollars to THE BISTOURY, we will furnish you our own journal, and "Good Health," for one year.

THE GAS CONSUMERS' GUIDE.—A popular Hand Book of instruction, on the proper management and economical use of gas, with a full description of gas meters, and directions for ascertaining the consumption, by meter, &c. Illustrated, 12 mo. cloth, $1.00, paper, 75 cts. Alexander Moore, Pub., Boston.

LITTLE CORPORAL.—This beautiful little magazine for children, continues to be as lively, chatty, and entertaining as ever. Is carefully edited, and is the most entertaining journal for the little folks that we know of. Price $1.50 a year. Published at Chicago, by John E. Miller. We will furnish it with the BISTOURY, at the same price.

A NEW BOOK BY DR. STORER.—We are in receipt of a work on the "Causation, Course and Treatment of Reflex Insanity in Women," by Horatio Robinson Storer, M. D., L. L. B., of Boston. It is received too late for a review in this number of The Bistoury. The reputation of the author is a sufficient guarantee to the profession, that the work is a valuable one.

A MODEL JOURNAL.—A model of typographical neatness, is Geo. P. Rowell & Co's Newspaper Reporter. It is a weekly magazine, printed upon beautifully tinted, heavy paper, and gives much useful information to Newspaper men. Messrs. Rowell & Co., have done much for the publishers of America, by furnishing their columns with advertisements from reliable parties, and doing the tedious work of collecting their dues for them, without expense or trouble to the publishers.

SATIN IN SOCIETY.—A real good, sensible book. Written in plain, comprehensive and chaste language, and should be a present from the father to every son, of fourteen years of age, in America. Fathers are too careless in instructing their sons upon the necessity for avoiding certain evil practices, which are by far too common among the youth of our land—leading them to early vices and shattered health. This book gives the desired advice and warning. *Every boy needs it.* Let him have it! C. F. Vent, Publisher, Cincinnatti, O. Price, $1.50

A MUCH NEEDED WORK.—Dr. Geo. H. Napheys has written another timely book, one that has been long needed by the youth of our land. It contains information that should be in the possession of every young man. Thousands have been ruined mentally and physically for the want of just such knowledge as this book affords. It is entitled "Councels on the Nature and Hygeine of the Masculine Function." Price $2,00, It will be sent by mail, post-paid, on receipt of price, by Hall Brothers, of this city.

### Answers to Correspondents.

DEAF MAN.—*Machias, N. Y.*—Do not wear those "ear-tubes"—they will render your hearing worse than it now is.

C. D.—*Huntingdon, Pa.*—You have Catarrh. Procure a nasal douche and use it with salt and water, as directed in a previous number of the BISTOURY.

Dr. C. H. W.—*Tecumseh, Neb.*—Flint's Practice of Med., Holmes' System of Surgery, Bedford's or Hodges' Obstetrics, is what you require.

Y. R. M.—*Waverly, N. Y.*—The treatment you propose for the cure of "film" on your horse's eye is absurd, even if you did find it in the *Rural New Yorker*. The idea of placing a lump of fresh butter in the ear of a horse to cure a "film" on his eye, is too ridiculous. Get your druggist to prepare the following:

Take Sulphate of Cadmium, 10 grains,
Rose water, 1 ounce,
Sulphate of atropia, 1 grain.

Mix, and apply it to your horse's eye, with a camel's hair brush, morning and evening. If this don't allay the inflammation and remove the scar, you can then try your fresh butter cure, and if it fails, hang a black cat by his left hind leg, with a black string, in the cellar, at midnight, when no moon is shining, and when he has ceased to squall, tickle the right hind leg of your horse with the extreme end of the cat's tail till the film disappears on the horse's eye. The last is as good a cure as the one *you* propose.

MRS. L. B.—*Dansville, N. Y.*—Tell your physician to procure you a Babcock Supporter. That is what you require.

DR. R.—*Brownsville, Tenn.*—Write to Geo. Tiemann & Co., 67 Chatham st., N. Y. They are the best instrument makers in this country, and can make your implement for you.

DR. S. R. M.—*Hannibal, Mo.*—Write to Wm. Wood & Co., New York, for the catalogue of medical books. You will there find what you need, with prices.

TRANSPLANTING THE SKIN.—Another triumph for surgery has occurred in the transplanting of healthy skin upon the surface of indolent or non-healing ulcers. In the large ulcers so often seen upon the limbs, and usually called "fever sores," and which are found so difficult to heal, the surgeon now covers over in a few days, by healthy skin, which grows from very small pieces taken from healthy parts, and transplanted upon the sore. A piece no larger than a mustard seed will cover a space as large as a silver quarter-dollar in four or five days time.

—Inclose your subscription in the envelope furnished for the purpose, and mail it to us at once, if you have not already done so.

### "HOW TO MAKE IT PAY."

Read the several premium lists in this number of the BISTOURY. We offer a splendid Sewing Machine for one hundred subscribers, Webster's Unabridged Dictionary for twenty-four subscribers, and will give the BISTOURY away by clubbing with other journals.

We offer very large *Cash* premiums to those who will work for our magazine. Our journal is so cheap—only fifty cents a year—that subscribers are easily secured for it. Write for specimen copies, and our Cash Premium List.

Address:

THE BISTOURY,
Elmira, N. Y.

### AN ELEGANT PREMIUM.

We are glad we hit upon the sewing machine premium. It has been the most successful one we have ever offered. Our lady canvassers are earning a sewing machine in two or three days time.

### For One Hundred Subscribers at fifty cents a subscriber, we will forward to any address, safely boxed, a

### "McLean & Hooper Sewing Machine,"

which we will guarantee to be equal, if not superior, to any machine in the market.

Only think of that! For fifty dollars we will forward one hundred copies of the BISTOURY and an elegant sewing machine!

The "McLEAN & HOOPER" makes the Elastic Lock Stitch," which will not rip, even if every third stitch be cut, and takes its thread directly from the spools—no shuttle being used. It will stitch, hem, fell, tuck, quilt, cord, bind, baste, braid, embroider and gather better than any machine in the market. As before stated, we will guarantee it to be a first-class machine in every particular.

We have had agents secure us one hundred subscribers in one single day. Our Magazine is cheap, useful, and a great help and convenience in any family. Send for sample copies—we will furnish them *free* to all who desire to canvass for it. Twenty-eight pages of reading matter in every number. Only fifty cents a year. Upon the receipt of fifty dollars we will ship, to any address, nicely boxed and packed, a "McLean & Hooper" family sewing machine, with extra needles, oil-can, screw-driver, gauge, and printed circulars complete. Be careful to give the Name, Post Office, County and State of every subscriber, plainly written. Send the money by draft, or post office order, to

THAD S. UPDEGRAFF, M. D.,
EDITOR OF BISTOURY,
ELMIRA, N. Y.

### ANOTHER PREMIUM.

WEBSTER'S DICTIONARIES.

We will cause to be forwarded, direct from the Publishers, by Express, one copy of "Webster's unabridged Dictionaries," (price, $12.00) 1840 pages, 3,000 illustrations, for *fifty* subscribers to the BISTOURY. Send us $25, with the addresses in full for fifty subscribers, and we will furnish the BISTOURY for one year, to every address, giving the dictionary as a premium, to the getter-up of the Club.

For *twenty-four* subscriber at fifty cents each, we will give as a premium, one copy of "Webster's National Pictorial Dictionary," (price $6,) 1040 pages and 600 illustrations.

It will be seen that we offer nothing but first-class premiums to those who work for our Magazine. We hope in this manner to double our circulation for volume VII. Send on the subscribers and secure the premiums offered.

### THE BISTOURY GIVEN AWAY.

OUR TERMS FOR CLUBBING WITH OTHER JOURNALS.

We will cause any of the publications below to be sent, together with THE BISTOURY, for one year, on the following terms:—

| | Pub. Price | with Bistoury. |
|---|---|---|
| Good Health, | $ 2.00 | 2.00 |
| Peterson's Ladies' Magazine. | 2.00 | 2.00 |
| Phrenological Journal, | 3.00 | 3.00 |
| Herald of Health, | 2.00 | 2.00 |
| Wood's Household Magazine, | 1.00 | 1.00 |
| "Little Corporal," | 1.50 | 1.50 |
| "Our Young Folks," | 2.00 | 2.00 |
| The Scientific American, | 3.00 | 3.00 |

Forward the price of any of the above publications to our address, and you will receive both journals regularly, for one year.

Address,

THE BISTOURY,
ELMIRA, N. Y.

